# Mechanisms of Antimicrobial Peptide Resistance in Gram-Negative Bacteria

**DOI:** 10.3390/antibiotics4010018

**Published:** 2014-12-25

**Authors:** Victor I. Band, David S. Weiss

**Affiliations:** 1 Department of Microbiology and Immunology, Emory University, Atlanta, GA 30329, USA; E-Mail: vband@emory.edu; 2 Yerkes Primate Research Center, Emory University, Atlanta, GA 30329, USA; 3 Emory Vaccine Center, Emory University, Atlanta, GA 30329, USA; 4 Division of Infectious Diseases, Department of Medicine, Emory University School of Medicine, Atlanta, GA 30329, USA

**Keywords:** antimicrobial peptide, AMP, CAMP, antibiotic resistance, Gram-negative

## Abstract

Cationic antimicrobial peptides (CAMPs) are important innate immune defenses that inhibit colonization by pathogens and contribute to clearance of infections. Gram-negative bacterial pathogens are a major target, yet many of them have evolved mechanisms to resist these antimicrobials. These resistance mechanisms can be critical contributors to bacterial virulence and are often crucial for survival within the host. Here, we summarize methods used by Gram-negative bacteria to resist CAMPs. Understanding these mechanisms may lead to new therapeutic strategies against pathogens with extensive CAMP resistance.

## 1. Introduction

Cationic antimicrobial peptides (CAMPs) have microbicidal properties towards a variety of pathogens including bacteria, viruses, fungi and parasites. They are a large and varied group of peptides produced by many organisms ranging from prokaryotes to vertebrates (over 1200 have been identified thus far) [1]. These peptides contain little consensus in their amino acid sequences, though they largely maintain certain key features: they are cationic, amphipathic and relatively hydrophobic [2]. These attributes are thought to allow CAMPs to interact with bacterial membranes which contain anionic head groups and hydrophobic fatty acid chains. The CAMPs then destabilize bacterial membranes, which can involve pore formation, leading to cell lysis [3]. Some CAMPs may also have intracellular targets whose inhibition can lead to disruption of cell wall, protein and nucleic acid synthesis, as well as the direct induction of cell death [4].

In addition to their roles in host defense, at least one class of CAMPs has been harnessed and used clinically to treat bacterial infections. The polymyxins, derived from the Gram-positive bacterium *Bacillus polymyxa*, are a class of antibiotics that have seldom been used due to nephrotoxic and neurotoxic side effects [5]. However, due to the catastrophic increase in antibiotic resistance, these drugs are increasingly used as last-line antibiotics to treat infections with multi-drug resistant Gram-negative pathogens [5]. Currently, two polymyxins are in use clinically, polymyxin B and colistin (polymyxin E).

While CAMPs can kill a variety of pathogens, Gram-negative bacteria represent a major target. Due to the intense pressure that CAMP-mediated killing exerts on bacteria, some species have evolved ways to resist the action of these antimicrobials. Resistance to CAMPs is thought to have a significant negative effect on the ability of the host to prevent and fight bacterial infections, and also threatens the utility of polymyxins in the clinic. Unfortunately, due to their recent increased use, resistance to polymyxins is already on the rise [6]. Taken together, the development of CAMP resistance likely allows Gram-negative bacteria to avoid killing by both the host immune system and polymyxin antibiotics. In this review, we will summarize the variety of methods used by Gram-negative pathogens to survive in the face of CAMPs, as well as the clinical implications of resistance to these peptides.

## 2. Surface Remodeling

**Figure 1:**
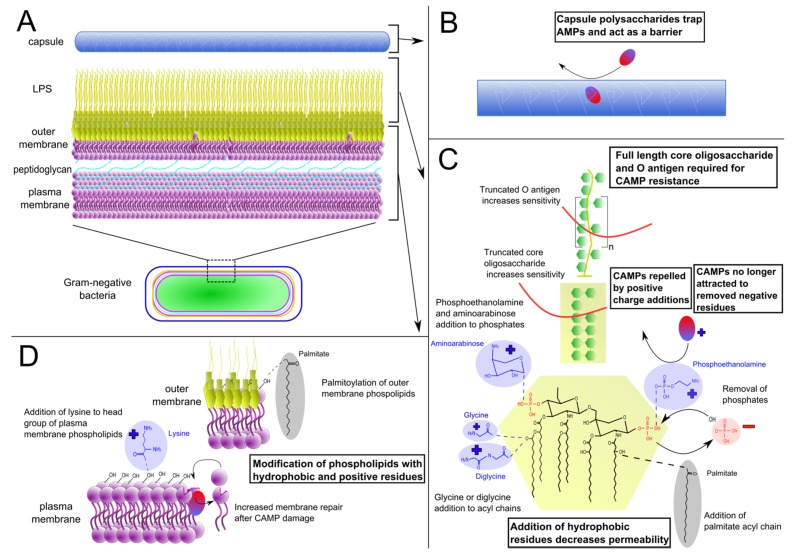
Bacterial surface modifications that enhance cationic antimicrobial peptide (CAMP) resistance. Gram-negative bacterial cell wall structure (**A**), with magnification of (**B**) capsule, (**C**) lipopolysaccharide, and (**D**) outer and plasma membranes. Lipopolysaccharide (LPS) structure varies greatly across species; depicted is a representative *E. coli* LPS structure, with modifications from various other species.

The outer surface of Gram-negative bacteria represents the first barrier to CAMPs and is therefore often modified to enhance resistance (Figure 1). One of the main ways in which killing by CAMPs can be avoided is through an increase in bacterial surface charge. Host CAMPs contain a region of highly positive charge and are attracted to negatively charged molecules, such as the surface of many bacteria. Thus, increasing surface charge prevents access of CAMPs to the vulnerable bacterial outer membrane [4].

The surface of Gram-negative bacteria is largely composed of the glycolipid lipopolysaccharide (LPS), serving as one of the initial barriers against extracellular stresses. Specifically, LPS is a major constituent of the outer leaflet of the outer membrane phospholipid bilayer, which envelops the peptidoglycan containing periplasm and the inner membrane (Figure 1A). It is comprised of the hydrophobic lipid A whose acyl chains insert into the membrane, the diverse core oligosaccharide, and the O-antigen comprised of repeating subunits (Figure 1C). In particular, the lipid A and core oligosaccharide often contain multiple negatively charged residues, such as phosphate groups.

### 2.1. Lipopolysaccharide Modifications

To mitigate the negative charge of LPS, numerous species of bacteria add positive residues to this structure, often to the lipid A. Common positively charged additions to LPS are cationic sugars. For example, the amine containing sugar aminoarabinose is added to a lipid A phosphate group in *P. aeruginosa* as well as *Salmonella typhimurium*, resulting in increased survival of both bacteria in the presence of polymyxin B [7,8]. Aminoarabinose is also present on the LPS of *Burkholderia* species [9]. However, this addition seems to be required for *Burkholderia* survival and so has not been directly linked to CAMP resistance. *Francisella novicida* adds another amine containing sugar, galactosamine, to its single lipid A phosphate group, similarly promoting polymyxin B resistance. Demonstrating the contribution of this modification to pathogenesis, deletion mutants lacking the galactosamine modification are highly attenuated, with a 5 log decrease in virulence in murine infections [10,11]. Increased CAMP resistance is also linked to cationic sugar addition in *Bordetella pertussis* and *Bordetella bronchiseptica*, in which glucosamine groups are added to both lipid A phosphates [10,12].

Other amine containing moieties, such as amino acids, are also added to the lipid A component of LPS to counteract its negative charge. For example, specific strains of *Vibrio cholerae*, the causative agent of the human disease cholera, add glycines to their lipid A [13]. While most strains of *Vibrio cholerae* are sensitive to CAMPs, the O1 El Tor strain, responsible for the current cholera pandemic [14], has a much higher level of resistance. Hankins *et al*. have shown that this strain of *V. cholerae* adds glycine and diglycine amino acid residues to lipid A acyl chains, increasing the net positive charge of LPS and the bacterial cell surface [13].

Phosphoethanolamine is another amine containing group that can be added to lipid A, as is the case in *Neisseria gonorrhoeae*, the causative agent of gonorrhea*.* This phosphoethanolamine residue is added to one of the phosphate groups of lipid A, under the control of the *lptA* gene that is required for its ability to resist CAMP-mediated killing [15]. Importantly, this increased resistance to CAMPs facilitates the establishment of a more severe disseminated form of gonorrheal infection [16]. Phosphoethanolamine addition to LPS also occurs in *Salmonella typhimurium* and in colistin-resistant strains of *A. baumannii* [17], where it increases resistance to polymyxin B [18].

In addition to adding positive charge to counteract the negative residues on LPS, some bacteria remove negative residues as an alternative mechanism of mitigating overall negative charge. The anionic phosphate groups of lipid A are major negative residues on LPS and are thus targets for removal. In *F. tularensis*, the 4' lipid A phosphate is removed by the phosphatase LpxF, leaving only one phosphate group on lipid A [19]. In *lpxF* deletion mutants that cannot remove the 4' phosphate, there is greatly increased susceptibility to polymyxin B, as well as the loss of lethality in a mouse intradermal infection [19,20]. Interestingly, the *lpxF* gene was exogenously expressed in *E. coli* whose lipid A normally has two phosphate groups. These modified *E. coli* lack a 4' lipid A phosphate and consequently display a >15-fold increase in polymyxin MIC [21]. The negatively charged phosphate groups on lipid A are a target for removal in many other pathogenic bacterial species, including *Porphyromonas gingivalis* [22], *Bacteroides fragilis* [23] and *Helicobacter pylori* [24]. Together, these data clearly demonstrate that CAMP resistance can be induced by removal of negatively charged lipid A residues.

Distinct from the alteration of charge, another strategy for generating CAMP resistance is to increase the hydrophobicity of LPS. Hydrophobic lipid chains, added to lipid A phosphates, the glucosamine backbone or existing acyl chains, serve to increase LPS saturation and decrease overall permeability, preventing CAMPs from inserting into the membrane [25]. In *Salmonella*, acyl chains are added to the glucosamine backbone and phosphates of lipid A by PagP [26]. *pagP* deletion mutants exhibited increased membrane permeability [26] and were nearly 4 times more susceptible to the antimicrobial peptide C18G (a synthetic CAMP derived from human platelet factor IV) [27]. Enhanced acylation of lipid A also occurs in *E. coli* and *Yersinia enterocolitica* [26].

Many of the LPS modifications described above are tightly regulated and induced upon exposure to CAMPs. The well characterized PhoPQ two component regulatory system of *Salmonella typhimurium* controls several modifications that lead to CAMP resistance [28]. It plays a major role in pathogenesis, since deletion mutants lacking this system had over a 4 log virulence defect during murine infection [29]. The fact that this regulatory system contributes to CAMP resistance suggests that avoiding killing by these antimicrobials is critical for virulence (Table 1). The sensor kinase PhoQ senses environmental stresses, such as low Mg^2+^ and Ca^2+^, as well as those encountered by bacteria within macrophage phagosomes, even directly detecting the presence of the CAMPs LL-37 and C18G [30], leading to activation of the response regulator PhoP [31]. PhoP subsequently activates the PmrAB two component system [31]. PmrAB signaling leads to modification of lipid A phosphates with aminoarabinose, increasing charge, and 2-hydroxy myristate, increasing hydrophobicity and decreasing permeability [32]. The PhoPQ and PmrAB systems play similar roles in *Pseudomonas aeruginosa* [33] and *Serratia marcescens* [34], while PmrAB functions in lipid A modification in *Acinetobacter baumannii* in the absence of PhoPQ [35]. These data highlight distinct ways that bacteria inducibly modify lipid A to resist CAMPs. However, lipid A is not the only portion of LPS that is a target for modification.

**Table 1:** Links between CAMP resistance and virulence of Gram-negative pathogens. Examples of CAMP resistance mechanisms that additionally have an impact on virulence. * Regulatory proteins shown to be responsible for CAMP resistance regulation, but regulate other processes as well.

Species	Modification	CAMP Resistance	Impact on Virulence	Ref.
*Brucella abortus*	LPS O antigen	Transposon mutants lacking O antigen show decreased survival to polymyxin B at 5–40 µg/mL	Transposon mutant unable to persist six weeks after mouse intraperitoneal infection	[36]
*Burkholderia cenocepacia*	LPS inner core oligosaccharide	*B. cenocepacia* require full length core oligosaccharide to grow in 100 µg/mL polymyxin B	Mutants with truncated core oligosaccharide were completely outcompeted by parent strain in rat lung infection model	[37]
*Legionella pneumoniae*	*rcp*, homolog of *pagP*, responsible for palmitoyl addition to lipid A	Mutants in *rcp* show 50% decrease in MIC to polymyxin B and synthetic CAMP C18G	Deletion mutants showed decreased survival in macrophages and were outcompeted by the parental strain in mouse lung infection	[38]
*Neisseria gonnorhoeae*	Mtr efflux pump	MICs are 8× higher for PG-1 and 30× higher for LL-37 in WT compared to *mtr* mutant	Deletion mutant completely outcompeted by WT after 3 day mouse genital tract infection	[39]
*Proteus mirabilis*	ZapA secreted metalloprotease	Purified ZapA readily degrades LL-37 and human beta-defensin-1 *in vitro*	4 log decrease in virulence in mouse urinary tract infection with ZapA mutant	[40]
*Pseudomonas aeruginosa*	AcrAB efflux pump	Mutant in a*crB* 10× more susceptible to CAMP-containing BALF, as well as diminished survival in 0.1 µg/mL polymyxin B, 30 µg/mL HNP-1, and 0.1 µg/mL HBD-1 + 2	1–3 log decrease in virulence of deletion mutant over 72 h mouse infection using a pneumonia model	[41]
	LasA cleavage and release of syndecan-1 from host immune cells	Shed syndecan-1 can bind Pro/Arg rich CAMPs	3 log decrease in virulence when syndecan-1 is absent in KO mouse lung infection, with 1/3 reduction in mortality	[42,43,44]
*Salmonella typhimurium*	Various	Transposon mutagenesis yielded 12 mutants that were susceptible to CAMP protamine at 1mg/mL	11 of 12 mutants with high protamine susceptibility had decreased virulence in mouse intragastric infection	[45]
	Aminoarabinose addition to lipid A through *pmrF*	*pmrF* deletion mutant unable to add aminoarabinose to lipid A, and is more sensitive to CAMPs	Mice orally infected with mutants had double the survival time as WT-infected mice. Competition infections with WT and deletion mutants show that CAMPs CRAMP and matrilysin alone not responsible for attenuation	[46]
	* PmrAB mediated addition of aminoarabinose to lipid A	Inactivation of *pmrA* results in 19× reduction in polymyxin B MIC, while overexpression results in 3× increase	*pmrA* deletion mutants show decreased lethality in mice by oral but not intraperitoneal infection	[8,47]
	* SlyA regulatory protein	*slyA* mutant is susceptible to 1 µg/mL polymyxin B, and SlyA protein binds to promoter of *ugtL* resistance gene	Deletion mutants have LD50 >4 log higher for oral infection and >5 log higher for peritoneal infection in mice	[48,49]
	* PhoP regulatory protein	Mutants increase sensitivity to human and rabbit neutrophil granules, as well as rabbit CAMP NP-1	Deletion mutants in *phoP* show 4 log reduction in virulence in mouse peritoneal model of infection, and *phoP*/*phoQ* deletion of *S. typhi* was a safe vaccine candidate in humans	[28,29,50]
*Yersinia enterocolitica*	Unspecified LPS modifications, possibly RosAB	Pathogenic *Y. enterocolitica* strains were more resistant to polymyxin B than non-pathogenic environmental strains when grown at 37 °C	Environmental strains not known to cause disease like the polymyxin resistant pathogenic strains	[51]

* Regulatory proteins shown to be responsible for regulation of antimicrobial peptide resistance, but regulate other processes as well.

In addition to lipid A modifications, the O-antigen and core sugars have also been implicated in CAMP resistance. In *Brucella abortus*, transposon mutants that lack O-antigen showed significantly decreased survival in polymyxin B and were attenuated in a mouse model [36]. Mutants in *Burkholderia cenocepacia* with a truncated core were unable to grow in high concentrations of polymyxin B as did the wild type strain, and they were additionally outcompeted in a lung infection model [37]. Full length core and O-antigen thus can significantly contribute to CAMP resistance and have an important impact on virulence.

It is important to note that many CAMP resistant bacteria use several of the strategies listed above to mitigate the negative charge of their LPS. For example, *Helicobacter pylori* not only decreases negative charge by removing a phosphate group, it also adds in its place a positively charged phosphoethanolamine [24], further increasing the charge of its lipid A. This results in extensive resistance to polymyxin B, with an MIC 25× higher than that of a deletion strain lacking these modifications. Many other Gram-negative bacteria use multiple strategies to mitigate the negative charge of LPS, and also modify other membrane components as well to further enhance CAMP resistance [52].

### 2.2. Phospholipid Modifications

In addition to LPS, phospholipids are the other major component of the Gram-negative outer membrane. Similar to LPS, phospholipids in the outer membrane can also be modified to increase CAMP resistance (Figure 1D). *S. typhimurium* uses its PhoPQ system to not only modify LPS, but also to modify phospholipids that reside in the outer membrane. PhoPQ-activated PagP adds palmitoyl groups to phospholipids, similar to its modification of lipid A described above. This leads to an increase in the levels of palmitoylated phosphatidylglycerols within the outer leaflet of the outer membrane, which are less polar and more hydrophobic than many other phospholipids in the outer membrane. Increased hydrophobicity in the outer membrane may decrease permeability, similar to the effect in lipid A palmityolation [53]. Therefore, localizing these modified phospholipids to the outer leaflet of the membrane results in increased CAMP resistance.

In addition, the inner membrane may be modified to increase CAMP resistance. Addition of lysine to phospholipids (lysylation) within the plasma membrane increases the charge of anionic phosphatidylglycerol to a cationic form, and thus is able to help repel cationic CAMPs and reduce their binding to the membrane. Though best studied in Staphylococcus aureus, these lysylated phospholipids are also present in Gram-negative species [54] including *Rhizobium tropici* [55] and *Caulobacter crescentus* [56].

It has also been suggested that the PagP protein mentioned above may act as part of an acute membrane repair response, facilitating rapid membrane repair after damage caused by CAMPs [53]. In addition, it has been hypothesized that one of the reasons that CAMPs do not efficiently damage eukaryotic host membranes is that eukaryotic cells have a much more robust form of membrane repair than bacteria [57]. Thus it is possible that bacteria with increased membrane repair capacity could survive higher concentrations of CAMPs, simply repairing the membrane as it is damaged. Dorschner *et al.* suggest that proteins involved in membrane repair are prime candidates for investigation of microbial resistance [58]. There is still a lack of concrete evidence demonstrating that this occurs, but membrane repair may be an important facet of CAMP resistance and warrants further investigation.

### 2.3. Capsule Production

Beyond alterations to the bacterial membranes, the outer surface of bacteria can be further modified to protect against CAMPs. The bacterial capsule is a protective layer external to the outer membrane that acts as an additional barrier and is comprised primarily of long chained repeating polysaccharides [59]. *Klebsiella pneumoniae* capsule provides increased resistance against cationic defensins, lactoferrins and polymyxins. Furthermore, there is a direct correlation between higher amounts of capsular polysaccharide, decreased levels of CAMPs binding to the outer membrane, and increased resistance to polymyxins [60]. Capsule-mediated resistance to CAMPs is likely critical for bacterial virulence during *in vivo* infection as an acapsular mutant was unable to cause pneumonia in a mouse model [61]. It should be noted, however, that the capsule can provide resistance to other immune pressures in addition to CAMPs, such as complement and phagocytosis, and thus the attenuation of the acapsular mutant is not necessarily due to a decrease in CAMP resistance.

In *Neisseria meningitidis*, capsule production was shown to increase resistance to the human CAMP LL-37 [62]. Survival in the presence of LL-37 was 100-fold lower in a deletion strain lacking capsule compared to wild-type. Furthermore, upon exposure to sublethal levels of LL-37, the capsule biosynthetic genes *siaC* and *siaD* were upregulated and contributed to increased capsule production [62].

In addition to those mentioned above, numerous Gram-negative species express a polysaccharide capsule. Further, *P. aeruginosa* has also been shown to use its capsule to resist CAMPs [63]. Taken together, the data described here illustrate how Gram-negative pathogens can use numerous modifications to LPS, phospholipids, and the production of a polysaccharide capsule to resist CAMPs and protect their membranes.

## 3. Biofilms

Bacteria can further resist CAMPs through their organization into specialized structures known as biofilms. In addition to free floating, planktonic bacterial populations, bacteria can form biofilms on diverse surfaces. These structures consist of sessile bacteria adhering to a surface in a highly organized manner that allows for circulation of nutrients [64]. Bacteria in a biofilm often secrete a slimy extracellular matrix that both aids in adherence to surfaces and acts as a barrier to outside stressors. This extracellular matrix can be composed of various compounds including cellulose, teichoic acids, proteins, lipids and nucleic acids [65]. Biofilms can form on environmental surfaces such as hospital equipment, allowing these populations to persist, and likely contributing to the growing problem of hospital-acquired infections. They can even form on ventilators and catheters, giving them access to mucosal sites in patients and further promoting their infectivity. Biofilms are also able to form on biological surfaces such as teeth or the respiratory tract, often facilitating the establishment of chronic infections [64].

The general organization of the bacteria and extracellular components contributes to the protection offered by the biofilm structure. As a biofilm matures, it progresses from a thin homogeneous structure to a thicker, more heterogeneous form that contains many substructures. These can include stacks of bacteria forming “mushroom” shaped structures [66]. This is observed in *Pseudomonas aeruginosa*, which forms biofilms that display exceptional resistance to CAMPs and antibiotics, in some cases over 1000 times as great as their planktonic form [67]. *Pseudomonas* biofilms contain a high level of the polysaccharide alginate, which is known to cause significant alterations to biofilm structure. A strain that overproduces alginate formed biofilms that were much thicker and more structurally heterogeneous, an architecture that acts as a more effective diffusion barrier to CAMPs [68]. Additionally, expression of *Pseudomonas* biofilm genes in *E. coli*, whose biofilms are normally flat and unstructured, resulted in the formation of biofilms with more complex architecture, correlating with increased resistance to the polymyxin antibiotic colistin. This increased resistance was not observed against other antibiotics such as ciprofloxacin, indicating that this protection may be specific to CAMPs [69]. Biofilm structure can vary greatly across different species and strains, which may account for some of the differences in CAMP susceptibility in various biofilms.

Specific components of the extracellular matrix have been shown to be critical for resistance to CAMPs. Anionic alginate in *P. aeruginosa* not only contributes to biofilm structure but can also bind to and induce conformational changes in invading CAMPs [70]. The CAMP-alginate complexes then oligomerize, hindering their ability to enter the biofilm [71]. Further, polysaccharides from biofilms of *K. pneumoniae* and *Burkholderia pyrrocinia* are able to bind and sequester CAMPs [72]. Adding these polysaccharides to *E. coli* increased its MIC to CAMPs LL-37, human beta defensin 3, and Bac7(1-35). Extracellular DNA also forms an integral component of *P. aeruginosa* [73] and *S. typhimurium* [74] biofilms, and can also contribute to CAMP resistance. The negative charge of DNA allows it to bind and sequester cations from the surrounding environment. This results in an environment with a low concentration of cations, which is an activating signal for the previously mentioned PhoPQ system. This therefore results in the activation of CAMP resistance genes via PhoPQ that lead to LPS and other modifications [73].

In addition to signaling by PhoPQ, biofilms have several other inducible defenses against CAMPs. *P. aeruginosa* encodes the inducible biofilm gene *psrA*, which has been linked with greatly increased levels of CAMP resistance [75]. This gene was upregulated 3-fold in the presence of the CAMP indolicidin. Deletion mutants lacking *psrA* were less able to form biofilms, and showed significantly increased killing when challenged with indolicidin or polymyxin B. Pamp *et al*. have shown that tolerance to colistin in *Pseudomonas* biofilms is due to metabolically active cells within the biofilm. While the less metabolically active cells in the biofilm were killed by colistin, a spatially distinct subset of more active cells were able to resist killing. These cells were able to upregulate PmrAB-regulated resistance genes responsible for lipid A modification [76]. Overall, biofilms confer bacteria with the ability to form a hardy structure that can withstand and resist destruction by high concentrations of CAMPs, as well as many other types of antimicrobials.

## 4. Efflux Pumps

Efflux pumps are complexes of mostly membrane bound proteins that move toxic compounds out of cells. Bacterial efflux pumps are active transporters, either directly requiring ATP or using an existing electrochemical potential gradient. These complexes play important roles in antibiotic resistance, as many bacteria use them to resist major classes of antibiotics, including fluoroquinolones, macrolides, tetracyclines, glycylcyclines, beta lactams and aminoglycosides [77]. In addition, bacterial efflux pumps contribute to colonization and persistence, likely in part by defending against host antimicrobials such as CAMPs [78]. Indeed, there are many examples of Gram-negative bacteria that use efflux pumps to increase survival and virulence *in vivo* even in the absence of antibiotics including *Salmonella typhimurium* [79,80], *Salmonella enteritidis* [81], *Enterobacter cloacae* [82], *Borrelia burgdorferi* [83], *P. aeruginosa* [84], *K. pneumoniae* [41], *V. cholerae* [85] and *N. gonnorhoeae* [39].

In addition to other resistance mechanisms described above, *K. pneumoniae* uses the AcrAB-TolC efflux pump system, known to mediate resistance against fluoroquinolones, to resist CAMPs. When the AcrB component of the efflux pump system was knocked out, mutant bacteria exhibited increased sensitivity to fluoroquinolones as well as polymyxin B [41]. The *acrB* mutant also exhibited 10-fold lower survival in bronchoalveolar lavage fluid, which contains many CAMPs, and specifically displayed increased sensitivity to the human alpha defensin HNP-1 as well as human beta defensins HBD-1 and HBD-2. Importantly, this increased susceptibility correlated with a 1–3 log attenuation of the mutant in a mouse pneumonia model [41].

Another pathogen that expresses efflux pumps to increase CAMP resistance is *Yersinia enterocolitica*. A human gut pathogen, *Y. enterocolitica* has a high level of resistance to human CAMPs, at least in part due to the action of the RosAB efflux pump system. A *rosAB* deletion mutant was more sensitive than wild-type to the CAMPs polymyxin B, cecropin P1 (produced in pig bladders) and melittin (found in bee venom) [86]. This pump acts as a potassium antiporter, using a potassium gradient that pumps K^+^ ions into the cell as it pumps out harmful CAMPs. Interestingly, the RosAB pump is activated at 37 °C and in the presence of CAMPs, similar to conditions encountered within the host during infection [86]. Under these conditions, pathogenic *Y. enterocolitica* strains are more resistant to CAMPs than non-pathogenic strains or a control *E. coli* strain [51]. While this was not explicitly shown to be due to the RosAB pump and could be due to another temperature regulated system, the data suggest that RosAB-mediated CAMP resistance is likely important for maintaining pathogenicity in *Y. enterocolitica*.

*N. gonorrhoeae* possess the Mtr (multiple transferrable resistance) efflux pump which facilitates resistance to numerous antimicrobials. This three protein system has been shown to pump out various hydrophobic compounds, such as bile salts and fatty acids, which can cause membrane damage. This pump also confers resistance to CAMPs as well. *mtr* deletion mutants had significant growth defects in the presence of PG-1, a protegrin produced by porcine macrophages [87], and the MIC of the human CAMP LL-37 and horseshoe crab-derived tachyplesin-1 were also reduced in the *mtr* deletion mutant. Thus, the Mtr efflux pump is able to recognize a variety of CAMP structures and remove them from the bacterial cell [87]. This efflux pump is highly relevant for *in vivo* survival; gonococci lacking *mtr* were completely outcompeted by the wild type strain in a competitive infection of the mouse genital tract [39] and this was correlated with the levels of CAMP resistance *in vitro* [88]. The closely related *Neisseria meningitidis*, which can cause meningitis in humans, also expresses the *mtr* efflux pump and it was similarly shown to contribute to CAMP resistance [89].

The RND family of efflux pumps in *Vibrio* species has a similar activity in mediating resistance to polymyxins and bile acids. *V. cholerae* has at least six loci that encode RND family proteins, including the VexB protein which can mediate CAMP resistance. When this protein is deleted from a virulent strain, the mutant bacteria exhibit increased susceptibility to polymyxin B as well as bile acids, which are found in the GI tract that *V. cholerae* infects. Further, this deletion mutant was unable to effectively colonize the gut of mice when compared to the wild-type strain [90]. The closely related *Vibrio vulnificus*, which can cause wound infections and sepsis, encodes a different efflux pump, TykA, which is responsible for resistance to the CAMPs protamine and polymyxin B [91].

Efflux pumps have been shown to be important for resistance to a wide range of antibiotics and there has been much interest in using efflux pump inhibitors to enhance antibiotic treatment [92]. However, the extensive evidence that these pumps can enhance CAMP resistance and play a role in virulence suggests that efflux pump inhibitors may also be used therapeutically to sensitize bacteria to innate immune defenses. Inactivating bacterial efflux pumps responsible for CAMP resistance could enhance the ability of the host CAMPs to clear infections, while at the same time increasing sensitivity to antibiotics.

## 5. Binding and Sequestering CAMPs

When confronted with a large concentration of CAMPs, some bacteria are able to bind and sequester these peptides so they cannot reach the bacterial membrane. One method for binding external CAMPs is through the release of negatively charged molecules that will attract these amphipathic antimicrobials. Negatively charged proteoglycans are found in abundance on the surface of fibroblasts and epithelial cells, and can be cleaved and released by bacterial enzymes at rates that exceed that of baseline release. For example, the connective tissue proteoglycan decorin is one of the major secreted products of human fibroblasts [93], and when incubated with *P. aeruginosa* or *P. mirabilis*, it is cleaved to release several products, including dermatan sulphate. This degradation occurs in the presence of bacteria conditioned media, purified *P. aeruginosa* elastase, or alkaline proteinase, even in the absence of fibroblast enzymes. This released dermatan sulphate was able to efficiently bind neutrophil derived α-defensin unlike the full length uncleaved decorin molecule. This free and soluble dermatan sulphate was able to nearly completely inhibit killing by defensins at concentrations 10 times above the MIC for *P. aeruginosa* [94].

Similarly, *P. aeruginosa* takes advantage of the release of the cell surface heparin sulfate proteoglycan syndecan-1. This proteoglycan is found on the surface of epithelial cells, and is shed during tissue injury as a soluble ectodomain. Incubating epithelial cells with cell culture supernatants from *P. aeruginosa* led to cleavage of syndecan-1 and release of its soluble ectodomain [42]. This activity was found to be dependent on the *P. aeruginosa* protein LasA, which is a known virulence factor and has been previously shown to modify other proteins. Shedded ectodomains of syndecan-1 are able to bind and interfere with the antimicrobial activity of CAMPs, specifically those that are Pro/Arg-rich like cathelicidins [42], likely due to charge based interactions. This was also demonstrated *in vivo*, with increased syndecan-1 shedding from epithelial cells during *P. aeruginosa* lung infection in a mouse model [43]. The virulence of the pathogen was dependent on syndecan-1 shedding, as there was a 3 log decrease in virulence if syndecan-1 was absent or rendered resistant to shedding [43]. Syndecan-1 ectodomains not only bind to CAMPs but can also bind and interfere with a range of other immune signaling molecules [42] such as cytokines and matrix metalloproteases [95]. It is not yet known the downstream effect that this binding would have on the greater immune response, but immune modulation in addition to direct interference with CAMPs may together account for the observed virulence decrease [43].

The fact that proteoglycans are able to interfere with host CAMP activity suggests that the bacterial capsule, which is rich in polysaccharides, may also be able to capture and sequester CAMPs [4]. Acapsular mutants often have decreased virulence *in vivo*, and *K. pneumoniae* acapsular mutants are more susceptible to α- and β-defensins [60]. This idea was further strengthened by evidence from Llobet *et al.*, showing that the anionic polysaccharide component (CPS) of the bacterial capsule is able to impart CAMP resistance in *K. pneumoniae* and *P. aeruginosa* [63]. Purified CPS was able to increase the resistance of acapsular mutants, and was shown to bind to soluble CAMPs in a charge-dependent manner. This resulted in fewer peptides reaching the surface of the bacteria. After exposure to CAMPs, these anionic polysaccharides are released by the bacteria to bind and sequester the antimicrobials [63]. It is possible that other encapsulated bacteria can use this mechanism to enhance CAMP resistance as well.

Another component that can be released to trap CAMPs is part of the bacterial cell membrane itself, in the form of enclosed vesicles budding off from the surface known as outer membrane vesicles (OMVs). OMV release is a normal part of bacterial cell growth [96] and may be used for a variety of processes such as toxin delivery [97]. In *E. coli*, membrane stress, especially from accumulation of proteins in the outer membrane, induces an increase in OMV formation. As the targets of CAMPs are bacterial membranes, CAMPs can be bound and sequestered in these vesicles, diverting them from the membranes of living bacteria. This notion is supported by the fact that mutants that over produce OMVs are 6-fold more resistant to killing by polymyxin B, while a mutant lacking vesicle release was 10-fold more susceptible [98]. *Vibrio cholerae* has adapted its OMV response to aid in CAMP resistance as well. In the presence of sublethal concentrations of polymyxin B, it was noted that OMVs released from the bacteria were larger and had altered protein content [99]. These OMVs were better able to protect against CAMPs, as co-incubating bacteria with them doubled the level of protection against LL-37 when compared to OMVs produced by bacteria in the absence of polymyxin B. The polymyxin B induced OMVs contained elevated levels of the protein Bap1, which was shown to mediate the increased CAMP protection by binding to but not degrading LL-37. Thus, OMV release can act as an inducible defense against CAMPs that can significantly increase levels of resistance.

## 6. Proteolytic Degradation

In addition to mechanisms to block access of CAMPs to bacteria, or pump them out of the cell, direct inactivation of these antimicrobials offers another means by which bacteria can combat them. As summarized below, diverse bacteria produce proteases that degrade CAMPs, an activity that is highly reliant upon the structural motifs of the target peptide. The human CAMP LL-37 is a linear, alpha helical peptide and is thus more susceptible to degradation by proteases than CAMPs with non-linear structures containing disulfide bonds such as defensins [100]. *P. aeruginosa* produces an elastase that is capable of rapidly degrading LL-37 *in vitro*, with its bactericidal activity completely inactivated within 1 hour [101]. Structural analysis showed that cleavage occurred at 4 peptide bonds all located within the regions of LL-37 that have bactericidal activity. Further, a *Proteus mirabilis* proteinase and *E. faecalis* gelatinase degrade and inactivate LL-37 *in vitro*, allowing for survival of bacteria in the presence of otherwise lethal doses of this antimicrobial [101]. In *S. typhimurium*, the omptin family protease PgtE degrades various alpha-helical CAMPs, including human LL-37 and its murine ortholog CRAMP [102]. Strains with deletions of this gene had 2-fold lower MICs to both CAMPs, while overexpression of *pgtE* increased the MIC by 8-fold. Interestingly, *pgtE* expression is regulated by PhoPQ, highlighting another example of the many CAMP resistance mechanisms controlled by this two-component system.

Even though linear CAMPs are quite sensitive to degradation by proteases, they can be shielded and protected by binding to proteins such as extracellular actin. *In vivo*, LL-37 can bind to released actin molecules, preventing the access of degradative proteases while still maintaining its antimicrobial activity [103]. High levels of extracellular actin were found in areas of cell necrosis, which often occurs at sites of infection [103]. Thus, linear CAMPs like LL-37 can be protected and rendered much less vulnerable to proteolytic degradation *in vivo* due to their complexing with other proteins.

Many non-linear CAMPs are more resistant to degradation than linear CAMPs. This is due at least in part to intramolecular disulfide bonds, such as those found in the defensins, which contain a canonical array of 6 disulfide linked cysteines [104]. These linkages create non-linear tertiary structure that is much more stable in the environment and resistant to protease degradation [105,106]. However, some bacteria have evolved proteases to degrade even these CAMPs with increased stability. The protein OmpT is another omptin family protease and contributes to CAMP resistance in *E. coli*. Stumpe *et al*. have shown that this outer membrane protein degrades the CAMP protamine [107] which is thought to conform to a nonlinear structure involving three disulfide bonds [108]. Both the OmpT and PgtE omptin proteins are present in *Shigella flexneri* and *Yersinia pestis*, suggesting that these pathogens may also use omptins to degrade CAMPs [109].

*B. cenocepacia* has two zinc dependent metalloproteases that have been shown to degrade CAMPs, ZmpA and ZmpB. Each of these proteins can degrade a variety of peptides, including a wide range of CAMPs. These proteins have distinct substrates, as only ZmpA can degrade linear LL-37, while only ZmpB degrades non-linear human beta defensin-1 [110]. Other CAMPs like protamine, elafin and SLPI (all of which are non-linear with disulfide bonds) were degraded by both, but they seemed to be digested into different fragments by each protease. These proteases are additionally important for the virulence of *B. cenocepacia,* since deletion mutants of each protease individually results in decreased lung pathology in a mouse infection model [111,112].

Along with its previously mentioned proteinase, *P. mirabilis* encodes the virulence factor ZapA that is involved in CAMP degradation. The ZapA protein is a secreted extracellular metalloprotease that is able to degrade a wide variety of targets, including host defense proteins such as immunoglobulins and complement components [113]. It is also able to target host CAMPs, including not only LL-37, but also disulfide bond containing defensin HBD-1. Proteolysis of LL-37 and HBD-1 by ZapA resulted in 6 and 9 fragments respectively, completely inactivating both proteins [113]. Importantly, absence of ZapA in *P. mirabilis* results in a 4 log decrease in bacteria in a mouse urinary tract infection model, suggesting that the degradation of host antimicrobials is vital to the virulence of this pathogen [40]. Many additional examples of bacteria directly degrading (or causing the degradation of) CAMPs likely exist, and presumably make important contributions to *in vivo* virulence.

Bacteria can also take advantage of host enzymes with CAMP-degrading activity. Reduced killing of *P. aeruginosa* by beta defensins in the broncho-alveolar fluid of cystic fibrosis patients has been shown to be due to the release of proteolytic cathepsins from macrophages, which are able to degrade host beta defensins. The release of cathepsins is due at least in part to the release of inflammatory mediators such as IL-13 and IFN-γ, which has been suggested to result from immune activation by LPS from *P. aeruginosa* and other commensal Gram negative bacteria [114]. Thus, *P. aeruginosa* is able to take advantage of the host immune response, facilitating the release of CAMP degrading enzymes.

## 7. Modulation of CAMP Expression

Cationic antimicrobial peptides are present in steady state at all mucosal sites of the body to prevent infection by invading microorganisms. However, in the context of an infection, CAMPs can also be upregulated to help fight the invading microbes. Stimulation of increased CAMP production occurs mainly through innate immune recognition of microbes [115] and subsequent signaling by Pattern Recognition Receptors (PRRs). For example, the host PRR Toll-like Receptor 4 (TLR4) recognizes and signals for a proinflammatory response, including CAMP induction, in response to LPS.

This also means that modulation of microbial components like LPS, such that they cannot be detected by host PRRs, can lead to decreased PRR signaling and thus lower levels of CAMPs. Modification of lipid A structure, which can greatly affect CAMP resistance (section 2.1), can also have a significant effect on TLR4 signaling. In *P. gingivalis*, removal of a single phosphate group from lipid A results in both increased resistance to polymyxin B [116] and reduced activation of TLR4 [117]. In addition, incorporation of a seventh acyl chain in *Salmonella* lipid A similarly results in increased CAMP resistance and decreased TLR4 signaling [118]. These examples highlight that modification of LPS structure can both directly (repel or prevent binding of CAMPs) and indirectly (reduced induction of CAMPs) lead to CAMP resistance.

Interestingly, LPS modifications that increase CAMP resistance in some bacteria actually increase detection by TLR4 [119]. For example, palmitoylation of lipid A in *P. aeruginosa* results in increased resistance to CAMPs but a more inflammatory LPS [120], indicating that CAMP resistance and evasion of TLR4 signaling do not always occur in tandem. It is possible that those modifications that are able to provide both increased CAMP resistance and evasion of TLR4 signaling are more beneficial to bacteria than modifications that provide only one of these attributes. Alternatively, increased inflammation may promote pathogenesis by some bacteria, and in these cases increased CAMP resistance as well as increased TLR4 signaling may be beneficial to the pathogen.

The link between tandem alterations of CAMP resistance and inflammatory signaling extends to other bacterial components and host receptors as well. Similar to LPS and TLR4, bacterial lipoproteins (BLP) are recognized by the host PRR Toll-like Receptor 2 (TLR2), leading to initiation of inflammatory signaling. In *Francisella novicida*, the CRISPR-Cas protein Cas9 plays a regulatory role in repressing the expression of a BLP [121]. This BLP repression leads to enhanced resistance to polymyxin B, as well as direct suppression of this TLR2 ligand and thus evasion of signaling (including CAMP induction) by this host receptor [122]. Many diverse PRRs detect bacteria, and avoiding recognition by these receptors could represent a broad and critical strategy to subverting the induction of CAMPs.

Another mechanism of limiting host inflammatory signaling, and thus CAMP induction, may be provided by the bacterial capsule. By facilitating resistance to CAMPs, the capsule prevents damage to bacterial membranes that contain activators of host signaling such as LPS and BLPs. As such, the capsule serves as a barrier to prevent the release of bacterial components that can be recognized by PRRs. This in turn prevents induction of higher levels of CAMPs. For example, in *P. aeruginosa*, the absence of capsule leads to an increased sensitivity to CAMPs as well as increased induction of beta defensins during murine infection [123]. Even further, the capsule polysaccharides of *P. aeruginosa* also activate the anti-inflammatory immune receptors CYLD and MKP-1, which results in the release of anti-inflammatory molecules that downregulate beta defensin production [123], illustrating yet another indirect mechanism by which the capsule facilitates resistance to CAMPs.

*Shigella*, which cause varying degrees of bacterial dysentery in children and adults, are able to downregulate the CAMP response of their human hosts through an as yet undetermined mechanism. Biopsies from *Shigella* infected colons show that there is a significant downregulation of transcripts encoding LL-37 and HBD-1 in epithelial cells, corresponding with decreased LL-37 and HBD-1 protein in the majority of the infected biopsies [124]. This downregulation was detected up to day 30, after which LL-37 levels began to increase above healthy control levels. Inhibition of LL-37 production was observed during *in vitro* infections of macrophage and epithelial cell lines, and was shown to be dependent upon *Shigella* plasmids within the host cells acting by an unknown mechanism [124]. It is known, however, that the MxiE protein controls the injection of plasmid encoded effectors into the host cell leading to this CAMP inhibition. Downregulation of CAMPs early in the infection likely enhances the ability of *Shigella* to adhere to mucosal surfaces and infect host epithelial cells [125].

In addition to the examples cited above, bacteria have a wide range of mechanisms of altering or avoiding the host immune response (for a review of these see Hornef *et al.* [126]). Many of these could result in decreased levels of CAMPs, which could aid in colonization and infection by bacteria. As CAMP levels are dynamic and intertwined with the overall immune response, it is likely that diverse strategies to limit immune signaling indirectly play important roles in CAMP resistance.

## 8. Relevance

We have highlighted in this review that resistance to CAMPs in Gram-negative bacteria is often a crucial virulence strategy. While there are abundant correlations between bacterial traits that increase CAMP resistance and affect virulence (Table 1), it is in most cases yet to be proven during *in vivo* infection that resistance to CAMPs facilitates pathogenesis. This is likely due in part to the possible redundancy of numerous host CAMPs, and therefore the difficulty in deleting a sufficient number of CAMPs from a host in many cases to observe robust phenotypes.

CAMP resistance also affects susceptibility to cationic antibiotics used in the clinic, the polymyxins. Resistance to host cationic antimicrobials may even be facilitated by resistance to polymyxins. Clinical strains of *A. baumannii* that are polymyxin resistant are significantly more resistant to both LL-37 and lysozyme [127]. This has also been demonstrated in *Enterobacter cloacae*, where colistin heteroresistant strains are resistant to lysozyme after initial treatment with colistin [128]. This cross resistance between polymyxins and host antimicrobials may interfere with the use of CAMPs as therapeutic tools, including not only polymyxins but many other CAMPs still in development [129]. This is a great concern in an era in which new antibacterials are not keeping pace with the emergence of resistance. This brings up the possibility of instead targeting CAMP resistance mechanisms therapeutically, which would theoretically increase their susceptibility to polymyxins in addition to reducing the virulence of pathogens.

## 9. Conclusions

Gram-negative pathogens use many diverse mechanisms to resist killing by cationic antimicrobial peptides. Bacteria can alter surface structures to repel CAMPs, establish biofilms to increase resistance, use efflux pumps to pump them out, sequester them, produce proteases to degrade them, or alter immune responses to prevent their induction. It is no surprise that these mechanisms (with the exception of immune modulation) are also used by bacteria to gain resistance to antibiotics and that many of the pathogens mentioned in this review are noted for their ability to resist antibiotics. In many instances, CAMP resistance increases the virulence of bacterial pathogens. The combination of increased antibiotic resistance and virulence due to CAMP resistance makes pathogens very dangerous in the clinical environment. Thus it is imperative to devise new ways to combat or reverse CAMP resistance in Gram-negative bacteria. Further understanding the mechanisms of CAMP resistance may be fruitful in deciphering new ways to combat highly virulent and antibiotic-resistant Gram-negative pathogens.
